# Bill of Fare

**Published:** 1883-04

**Authors:** 


### BILL OF FARE FOR EACH DAY OF THE WEEK.

### SUNDAY.

**BREAKFAST.**
Cream cod fish, or fish cakes.
Eggs.
Corn bread,
Coffee.

**DINNER.**
Soup,
Roast beef, or turkey,
Cranberry Sauee,
Mashed potatoes,
Corn, Asparagus,
Tomatoes,
Salad of lettuce,
Custard meringue,
Fresh ripe fruit,
Nuts and raisins.

**TEA.**
Chipped beef,
Bread and butter,
Fruit, stewed or canned,
Cake.

### MONDAY.

**BREAKFAST.**
Oat meal,
Mutton chops,
Potato cakes,
Muffins,
Coffee.

**DINNER.**
Cold roast beef, or beef stew with riceand curry.
Boiled potatoes,
Lima beans,
Rice pudding, with jelly.

**TEA.**
Ham omelet,
Toast,
Fresh or canned peaches,
Cake.

### TUESDAY.

**BREAKFAST.**
Hominy,
Veal cutlets,
Hashed potatoes,
Rolls,
Coffee.

**DINNER.**
Soup,
Roast veal, or roast pork,
Baked potatoes,
Potato salad,
Peas,
Bread pudding, with cream sauce.

**TEA.**
Scalloped oysters,
Tea biscuit and honey,
Apple sauce with lemon.
Sponge cake.

### WEDNESDAY.

**BREAKFAST.**
Oat meal,
Ham and eggs,
Toast,
Fried potatoes,
Coffee.

**DINNER.**
Roast or boiled mutton,
Apple sauce,
Stewed onions,
Mashed potatoes,
Baked tomatoes,
Cake and fruit,
Nuts and raisins.

**TEA.**
Cold mutton,
Milk toast,
Stewed plums,
Cake.

### THURSDAY.

**BREAKFAST.**
Corn bread,
Fried liver, with bacon,
Potato cakes,
Coffee.

**DINNER.**
Beef, pot roast,
Maccaroni with cheese,
Boiled potatoes,
Corn and tomatoes,
Cottage or cabinet pudding.

**TEA.**
Broiled mackerel,
Tea biscuit,
Honey or jam,
Jelly cake.

### FRIDAY.

**BREAKFAST.**
Hominy,
Liver hash,
Stewed beef,
Graham gems,
Coffee.

**DINNER.**
Baked fish, garnished with [illegible] lemon,
Beef steak.
Baked potatoes,
Beets and turnips,
Boiled rice,
Tapioca, or birds' nest pudding.

**TEA.**
Omelet,
Muffins,
Stewed prunes,
Cake.

### SATURDAY.

**BREAKFAST.**
Rice cakes,
Pork chops, or beef steak,
Eggs,
Rolls,
Coffee.

**DINNER.**
Baked pork and beans,
Corned beef and vegetables,
Potato salad,
Baked tomatoes.
Boiled apple pudding, or Indian pudding.

**TEA.**
Broiled or fried chicken,
Biscuit,
Marmalade, or
Preserved ginger,
Cake.

